# The joint lasso: high-dimensional regression for group structured data

**DOI:** 10.1093/biostatistics/kxy035

**Published:** 2018-09-05

**Authors:** Frank Dondelinger, Sach Mukherjee, The Alzheimer’s Disease Neuroimaging Initiative

**Affiliations:** 1 Lancaster Medical School, Lancaster University, Furness College, Bailrigg, Lancaster, UK; 2 Statistics and Machine Learning, German Center for Neurodegenerative Diseases (DZNE), Sigmund-Freud-Straße 27, Bonn, Germany

**Keywords:** Group-structured data, Heterogeneous data, High-dimensional regression, Penalized regression, Information sharing

## Abstract

We consider high-dimensional regression over subgroups of observations. Our work is motivated by biomedical problems, where subsets of samples, representing for example disease subtypes, may differ with respect to underlying regression models. In the high-dimensional setting, estimating a different model for each subgroup is challenging due to limited sample sizes. Focusing on the case in which subgroup-specific models may be expected to be similar but not necessarily identical, we treat subgroups as related problem instances and jointly estimate subgroup-specific regression coefficients. This is done in a penalized framework, combining an $\ell_1$ term with an additional term that penalizes differences between subgroup-specific coefficients. This gives solutions that are globally sparse but that allow information-sharing between the subgroups. We present algorithms for estimation and empirical results on simulated data and using Alzheimer’s disease, amyotrophic lateral sclerosis, and cancer datasets. These examples demonstrate the gains joint estimation can offer in prediction as well as in providing subgroup-specific sparsity patterns.

## 1. Introduction

High-dimensional regression has been well studied in the case where all samples can reasonably be expected to follow the same model. However, in several current and emerging applications, observations span multiple subgroups that may not be identical with respect to the underlying regression models. In biomedical problems, sets of samples representing, for example, disease subtypes may differ with respect to underlying biology, and therefore have different relationships between observed features and a response of interest.

A topical example, to which we return below, is in the study of neurodegenerative diseases such as Alzheimer’s disease (AD). In AD, there is as yet no disease-modifying therapy and no reliable way to predict future disease course (Ewers *and others*, 2011). The latter is important both for targeting candidate therapies at an early stage of the disease and for more general screening purposes, for example to target preventative interventions. The Alzheimer’s Disease Neuroimaging Initiative (ADNI) is a collaborative, open science initiative for AD. The ADNI studies include cognitively normal (CN) subjects, as well as subjects with AD and with mild cognitive impairment (MCI). It is likely that patterns of association between various high-dimensional data and phenotypes of interest (such as cognitive scores) differ between these study groups due to differences between the respective subpopulations and underlying biological factors. If so, a single regression model imposed on all of the data may be mis-specified, possibly severely. An alternative would be to build a separate model for each group. However, the sample size per group is then necessarily strictly smaller than the total sample size, making estimation challenging, especially when the number $p$ of features is large. Then, predictive ability and, just as important, the ability to efficiently estimate subgroup-specific influences or sets of influential factors may be compromised. This general situation is increasingly common in the emerging area of stratified medicine, where studies are designed to span one or both of disease stages and disease subtypes (if not multiple different diseases). At the same time emerging studies are increasingly high-dimensional in terms of the total number of features of potential relevance. These factors motivate a need for flexible models that are statistically efficient and scalable enough for practical application in high-dimensional biomedical studies.

Here, we focus on the specific case of high-dimensional regression in group-structured settings. In particular, we consider linear regression in the scenario in which the same set of $p$ features or predictors is available in each of $K$ subgroups. That is, we consider subgroup-specific linear regression problems indexed by $k$, each with subgroup-specific sample size $n_k$, a response vector $y_k$ of length $n_k$, a $n_k \times p$ feature matrix $X_k$ and a $p$-vector $\beta_k$ of regression coefficients. The problem we address is estimating the regression coefficients $\beta_1 \ldots \beta_K$.

We propose an approach to jointly estimate the regression coefficients that induces global sparsity and encourages similarity between subgroup-specific coefficients. We consider the following penalized formulation and its variants

$$\begin{eqnarray*}\hat{B} & = & \underset{B=[{{\beta }_{1}}\ldots {{\beta }_{k}}]}{\mathop{\arg \min }}\,\ \sum_{k=1}^K \left\{ \frac{1}{n_k} \|y_k - X_k \beta_k\|_2^2 + \lambda \| \beta_k \|_1 + \gamma \sum_{k' > k} \tau_{k, k'} \| \beta_k - \beta_{k'} \|_2^2 \right\}\end{eqnarray*}$$

where $B=[\beta_1 \ldots \beta_K]$ is a $p \times K$ matrix that collects together all the regression coefficients, $\| \cdot \|_q$ denotes the $\ell_q$ norm of its argument and $\lambda, \gamma, \tau$ are tuning parameters. The last term is a fusion-type penalty between subgroups; note that the difference is taken between entire vectors of subgroup-specific coefficients. An $\ell_2$ fusion penalty is shown above, although other penalties may be used; in this article, we also consider an $\ell_1$ variant. The parameters $\tau_{k,k'}$ allow for the possibility of controlling the extent to which similarity is encouraged for specific pairs of subgroups. In contrast to simple pooling, our approach, which we name the joint lasso, allows subgroups to have different sparsity patterns and regression coefficients, but in contrast to the subgroup-wise approach it takes advantage of similarities between subgroups.

The joint lasso shares similarities with both the group lasso (Yuan and Lin, 2006) and the fused lasso (Tibshirani *and others*, 2005) but differs from both in important ways. In contrast to the group lasso, we consider subgroups of samples or observations rather than groups of coefficients and in contrast to the fused lasso, we consider fusion of entire (subgroup-specific) coefficient vectors, rather than successive coefficients under a pre-defined ordering. Obozinski *and others* (2010) showed how the group lasso could be used in subgroup-structured settings, essentially by considering the global problem and defining groups (in the group lasso sense) corresponding to the same features across all subgroups. This means that each feature tends either to be included in all subgroup-specific models or none. In contrast, the joint lasso allows subgroups to have different sparsity patterns, whilst pulling subgroup-specific coefficients together and inducing global sparsity. Our work is also similar in spirit to recent work concerning joint estimation of graphical models over multiple problem instances (Danaher *and others*, 2014; Oates *and others*, 2014, 2015).

We show empirical results in the context of two neurodegenerative diseases—AD and amyotrophic lateral sclerosis (ALS). The methods we propose are general and we show also an application to cancer cell line data (see below for full details of the applications and data). The responses concern disease progression in AD and ALS and therapeutic response in cancer cell lines. In the AD and ALS examples, subgroups are based on clinical factors, while in the cancer data they are based on the tissue type of the cell lines.

Across the three examples, data types include genetic, clinical, and transcriptomic variables. We find that the joint lasso can improve performance relative to pooling or subgroup-wise analysis. Importantly, in cases where pooling or subgroup-wise analyses do well (perhaps reflecting a lack of subgroup structure or insufficient similarity, respectively) our approach remains competitive. This gives assurance that penalization is indeed able to share (or not share) information appropriately in real-world examples. We emphasize that the goal of the empirical analyses we present is not to give the best predictions possible in these applications, but rather to better understand the potential of joint estimation in group-structured problems.

## 2. Methods

### 2.1. Notation

Each subgroup $k \in \{1 \ldots K \}$ has the same set of $p$ features, but subgroup-specific sample size $n_k$. Total sample size is $n=\sum_{k =1}^K n_k$. For subgroup $k$, $X_k$ is the $n_k \times p$ feature matrix and $y_k$ the corresponding $n_k \times 1$ vector of observed responses. Subgroup-specific regression coefficients are $\beta_k \in \mathbb{R}^p$. Where convenient we collect all regression coefficients together in a $p \times K$ matrix $B = [\beta_1 \ldots \beta_K]$ and accordingly we use $\beta_{j,k}$ to denote the coefficient for feature $j$ in subgroup $k$.

### 2.2. Model formulation

We seek to jointly estimate the regression coefficients $B = [\beta_1 \ldots \beta_K]$ whilst ensuring global sparsity and encouraging agreement between subgroup-specific coefficients. We propose the criterion

(2.1) \begin{equation*}\label{eq:model_l2_fusion}\hat{B} = \underset{B= [\beta_1 \ldots \beta_K]}{\mathop{\arg \min }}\,\ \sum_{k=1}^K \left\{\frac{1}{n_k} \|y_k - X_k \beta_k\|_2^2 + \lambda \| \beta_k \|_1 + \gamma \sum_{k' > k} \tau_{k, k'} \| \beta_k - \beta_{k'} \|_2^2 \right\} \end{equation*}

and a variant with an $\ell_1$ norm in the last term

(2.2) \begin{equation*}\label{eq:model_l1_fusion} \hat{B} = \underset{B}{\mathop{\arg \min }}\,\ \sum_{k=1}^K \left\{\frac{1}{n_k} \|y_k - X_k \beta_k\|_2^2 + \lambda \| \beta_k \|_1 +\gamma \sum_{k' > k} \tau_{k, k'} \| \beta_k - \beta_{k'} \|_1 \right\}\! .\end{equation*}

Here, $\lambda, \gamma, \tau$ are tuning parameters. The role of the last term is to encourage similarity between subgroup-specific regression coefficients. The special case $K=1$ recovers the classical lasso (applied to all data pooled together). The tuning parameters $\tau_{k,k'}$ give the possibility of controlling the extent of fusion between specific subgroups. By default all $\tau$’s are set to unity (“unweighted fusion”), but they can also be set to specific values as discussed below (“weighted fusion”). In the above formulation, we assume that $y_k$ and $X_k$ have been standardized (at the subgroup level) so that no intercept terms are required. Note that the regularization parameters $\lambda, \gamma$ are the same across subgroups. The $\frac{1}{n_k}$ factor in the squared loss term corrects for subgroup size, to allow for the same amount of regularization across subgroups.

The difference between the two variants is that the first, $\ell_2$ fusion encourages similarity between subgroup-specific coefficients, while the second $\ell_1$ version allows for exact equality. The $\ell_2$ formulation has the computational advantage that the fusion part of the objective function becomes continuously differentiable, and the estimate of the objective function at each step can be obtained by soft-thresholding, analogously to co-ordinate descent for regular lasso problems. In the $\ell_1$ formulation on the other hand, the fusion constraint is only piece-wise continuously differentiable, leading to a more difficult optimization problem (see below).

### 2.3. Comparison with group and fused lasso

It is instructive to compare our formulation to the group lasso and the fused lasso, and to highlight the important ways in which it differs from both.

The original group lasso (Yuan and Lin, 2006) was designed to consider groups of features within a single regression problem. Let $X$ be the feature matrix and $y$ the vector of responses in a standard regression problem. Letting $l \in \{1 \ldots L \}$ index groups of features, the group lasso criterion is

(2.3) \begin{eqnarray*}\hat{\beta} & = & \underset{\beta}{\mathop{\arg \min }}\,\ \| y - \sum_{l=1}^L X^{(l)} \beta^{(l)} \|_2^2 + \sum_{l=1}^L \lambda_l \|\beta^{(l)} \|_2\end{eqnarray*}

where $X^{(l)}$ is the submatrix of $X$ corresponding to the features in group $l$, $\beta^{(l)}$ the corresponding regression coefficients and $\lambda_l$ a tuning parmeter. The penalty tends to include or exclude all members of a group from the model, i.e. all coefficients in a group may be set to zero giving groupwise sparsity.

In our setting, the subgrouping is over subsets of samples, and not over groups of features. It would therefore seem that there is little relationship between the joint lasso and the group lasso. However, in Obozinski *and others* (2010), a group lasso-like criterion was used for estimation in the multiple subgroup setting. Using the sum squared error as the loss function, the model in equation (2) of Obozinski *and others* (2010) becomes:

(2.4) \begin{eqnarray*}\hat{\beta} & = & \underset{B= [\beta_1 \ldots \beta_K]}{\mathop{\arg \min }}\,\ \sum_{k=1}^K \| y - X_k \beta_k \|_2^2 + \lambda \sum^p_{j=1} \|\beta_{j,:} \|_2\end{eqnarray*}

where $\beta_{j,:}$ denotes a vector of coefficients for a single feature $j \in \{ 1 \ldots p \}$ across all $K$ subgroups. This formulation encourages features to either be included in all the subgroup-specific models or none. However, unlike the joint lasso model, this formulation does not encourage similarity across subgroups among the non-zero covariates.

The fused lasso (Tibshirani *and others*, 2005) is also aimed at a single regression problem, but assumes that the features can be ordered in such a way that successive coefficients may be expected to be similar. This leads to the following criterion

(2.5) \begin{equation*}\label{eq:sim_fused_1d} \hat{\beta} = \underset{\beta}{\mathop{\arg \min }}\,\ \|y - X \beta \|^2_2 + \lambda \|\beta \|_1 + \gamma \sum_{i=1}^{p-1} \|\beta_i - \beta_{i+1} \|_1\end{equation*}

where $\lambda,\gamma$ are tuning parameters and we have assumed that the features are in a suitable order. The final term encourages similarity between successive coefficients. Efficient solutions for various classes of this problem exist (e.g. Hoefling, 2010; Liu *and others*, 2010; Ye and Xie, 2011).

Our approach shares the use of a fusion-type penalty, but focuses on a different problem, namely that of jointly estimating regression coefficients across multiple, potentially non-identical, problems. Accordingly, the joint lasso penalty encourages agreement between entire coefficient vectors from different subgroups and does not require any ordering of features.

### 2.4. Setting the tuning parameters $\tau$

For weighted fusion, the parameters $\tau_{k,k'}$ could be set by cross-validation but this may be onerous in practice. As an alternative we consider setting $\tau_{k,k'}$ using a distance function $d(k, k')$ based on the features. The idea is to allow more fusion between subgroups that are similar with respect to $d$, while allowing the $\tau_{k,k'}$ to be set in advance of estimation proper. However, this assumes that similarity in the features reasonably reflects similarity between the underlying regression coefficients, which may or may not be the case in specific applications.

We consider two variants. The first sets $d(k,k') = \| \mu_k - \mu_{k'} \|_2$ where $\mu_k, \mu_{k'}$ are the sample means of the features in the subgroups $k,k'$, respectively (we assume the data have been standardized). The second approach additionally takes the covariance structure into account by using the symmetrised Kullback–Leibler (KL) divergence, i.e. $d(k,k') = \frac{1}{2}(\mathrm{KL}(\hat{p}_k \| \hat{p}_{k'}) + \mathrm{KL}(\hat{p}_{k'} \| \hat{p}_k))$, where $\hat{p}_k, \hat{p}_{k'}$ are estimated distributions over the features in the subgroups $k,k'$ respectively and $\mathrm{KL}(p \| q)$ is the KL-divergence between distributions $p$ and $q$. In practice, this requires simplifying distributional assumptions. Below we use multivariate Normal models for this purpose, with the graphical lasso (Friedman *and others*, 2008) used to estimate the $\Sigma_k$’s. For both approaches, we set $\tau_{k,k'} = 1 - d(k,k')/d_{max}$, with $d_{max}$ the largest distance between any pair of groups $k$, $k'$ (this scales $\tau$ to the unit interval).

### 2.5. Optimization

We describe a co-ordinate descent approach for optimizing equation (2.1). While it is possible to derive a block coordinate descent approach for equation (2.2) (e.g. following Friedman *and others*, 2007), this is generally inefficient for the high-dimensional problems that we consider. Instead, we will describe an optimization procedure based on a proximal gradient approximation derived in Chen *and others* (2010).

#### 2.5.1. Co-ordinate descent for $\ell_2$ fusion.

The $\ell_2$ fusion penalty is continuously differentiable and we can obtain the optimal value for $\hat{\beta}_{j,k}$ in equation (2.1) at each step by first calculating optimal values without the lasso penalty:

(2.6) \begin{equation*} \label{eq:l2_update} \hat{\beta}^*_{j,k} = \frac{x_{j,k}^T (y_k - X_{-j,k}\beta_{-j,k}) + n_k \gamma \sum_{k' \neq k} \tau_{k,k'} \beta_{j,k'}} {x_{j,k}^T x_{j,k} + n_k \gamma \sum_{k' \neq k}\tau_{k,k'}}\end{equation*}

Then $\hat{\beta}_{j,k}$ can be obtained by soft-thresholding on $\hat{\beta}^*_{j,k}$. The procedure is summarized in Algorithm 1.

**Algorithm 1:**
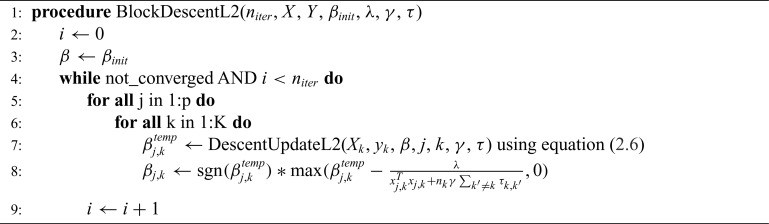
Block co-ordinate descent for the joint lasso

While Algorithm 1 is easy to understand and implement, a naive implementation in most programming languages will be still be slow due to the need for an inner for-loop over $p$, where $p$ can be in the tens of thousands for the kinds of problems we will consider. In order to efficiently optimize $B$, we reformulate (2.1) as a classical lasso problem and apply the glmnet software (Friedman *and others*, 2010). We transform the sum in first part of the objective into matrix form $y_{flat} - X_{diag}b_{flat}$ by defining $X_{diag}$ as a block-diagonal $n \times pK$ matrix with $X_k$ along the diagonals. The vector $b_{flat}$ is a flattened version of $B$ with stacked $\beta_k$ vectors, and similarly for $y_{flat}$. So we have:

$$\begin{equation*}{{X}_{diag}}=\left( \begin{matrix}   {{X}_{1}} & {} & {}  \\   {} & \ddots  & {}  \\   {} & {} & {{X}_{K}}\quad   \\\end{matrix} \right)\,\,\,\,\,\,\,\,\,\,\,\,\,\,\,\,{{b}_{flat}}=\left( \begin{matrix}   {{\beta }_{1}}  \\   \vdots   \\   {{\beta }_{K}}  \\\end{matrix} \right){{y}_{flat}}=\left( \begin{matrix}   {{y}_{1}}  \\   \vdots   \\   {{y}_{K}}  \\\end{matrix} \right)\end{equation*}$$

Now we move the $\ell_2$ fusion penalty into the first squared term by defining the augmented matrix $X^{aug}_{diag}$, and augmented vector $y^{aug}_{flat}$, such that

(2.7) \begin{equation*}\label{eq:model_l2_fusion_glmnet}\hat{b}_{flat} = \underset{b_{flat}}{\mathop{\arg \min }}\,\ \|y^{aug}_{flat} - X^{aug}_{diag} b_{flat}\|_2^2 + \lambda \| b_{flat} \|_1\end{equation*}

where

$$\begin{equation*}X_{diag}^{aug}=\left( \begin{matrix}   {{X}_{diag}}  \\   \Gamma   \\\end{matrix} \right)\,\,\,\,\,\,\,\,\,\,\,\,\,\,y_{flat}^{aug}=\left( \begin{matrix}   {{y}_{flat}}  \\   {\vec{0}}  \\\end{matrix} \right)\end{equation*}$$

with $\Gamma$ a $pK(K-1)/2 \times pk$ matrix encoding the pair-wise fusion constraints, and $\vec{0}$ a $pK(K-1)/2 \times 1$ vector of zeros. Each block $\Gamma_{k,k'}$, $k,k' \in [1,K], k<k'$ of $p$ rows of $\Gamma$ corresponds to the fusion constraint between two coefficient vectors $\beta_k$ and $\beta_{k'}$, with:

(2.8) \begin{equation*}\Gamma_{k,k'}(l,m) = \begin{cases} \gamma \tau_{k, k'} & \quad \text{if } l=p(k-1)+m \\ -\gamma \tau_{k, k'} & \quad \text{if } l=p(k'-1)+m\\ 0 & \quad \text{otherwise.} \end{cases}\end{equation*}

We can see that (2.7) is a classical lasso problem, to which glmnet can be directly applied.

#### 2.5.2. Proximal-gradient approach for fused L1 penalty.

Optimizing equation (2.2) by block gradient descent, while possible, is highly inefficient due to having to deal with the discontinuities in the objective function space. In Chen *and others* (2010), the authors describe a proximal relaxation of this problem that introduces additional smoothing to turn the objective function $f_{L1}(B)$ into a continuously differentiable function $f^{\mu}_{L2}(B)$. Chen *and others* (2010) deal with the multi-task regression setting (with common $X$ for each task); it is straightforward to adapt their procedure for the subgroup regression setting with different $X_k$ per subgroup.

It is notationally convenient to first introduce a graph formulation of the fusion penalties. We will think of the fusion constraints in terms of an undirected graph $G=(V,E)$ with vertex set $V = \{ 1 \ldots K \}$ corresponding to the subgroups and edges between all vertices. Then the $\ell_1$ penalised objective function can be written as:

(2.9) \begin{equation*} \label{eq:sim_fused_l1}f_{L1}(B) = \sum_k \left\{\frac{1}{n_k} \|y_k - X_k \beta_k\|^2_2\right\} + \| BC \|_1\end{equation*}

where the last term includes both sparsity and fusion penalties, via the matrix $C=(\lambda I_K, \gamma H)$, with $I_K$ the identity matrix of size $K$, $C$ a $K \times |E|$ matrix ($|E| = {\binom{K}{2}}$ in this case), and where for any $k \in V$ and $e = (m,l) \in E$:

(2.10) \begin{equation*}H_{k,e} = \begin{cases} \tau_{m,l} & \quad \text{if } k=m \\ -\tau_{m,l} & \quad \text{if } k=l\\ 0 & \quad \text{otherwise.} \end{cases}\end{equation*}

Note that unlike in Chen *and others* (2010), we require the explicit sum over $k$ in the objective to account for different sample sizes $n_k$ in different groups^1^.

The graph formulation allows for zero edges by setting $\tau_{k,k'}$ to zero. We have implicitly assumed in the formulation of (2.1) and (2.2) that the relationship between subgroups is represented by an undirected graph. However, (2.9) is completely general, and it would be straightforward to incorporate a directed graph in our model. We have not pursued this avenue here, as there is no reason to suspect directionality in the subgroup relationships for the applications we consider below, and including directionality would double the number of tuning parameters $\tau_{k,k'}$ that need to be considered.

Following Chen *and others* (2010), we can introduce an auxiliary matrix $A \in \mathcal{Q}=\{A'| ~ \|A'\|_{\infty} \leq 1,$$A' \in \mathcal{R}^{p \times (K + |E|)}\}$. Because of duality between $\ell_1$ and $\ell_\infty$, we can write $\|BC\|_1 = \max_{\|A\|_\infty \leq 1} \langle A, BC \rangle$. A smooth approximation of $\|BC\|_1$ is then obtained by writing:

(2.11) \begin{equation*}\label{eq:smooth_approx}f_\mu(B) = \max_{\|A\|_\infty \leq 1} \langle A, BC \rangle - \mu d(A)\end{equation*}

where $\mu$ is a positive smoothness parameter, and $d(A) \equiv \frac{1}{2}\|A\|^2_F$, with $\|\cdot\|_F$ the Frobenius norm. They show that for a desired accuracy $\epsilon$, we need to set $\mu=\frac{\epsilon}{p(K+|E|)}$. Theorem 1 in Chen *and others* (2010) gives the gradient of $f_\mu(B)$ as $\Delta f_\mu(B) = A^*C^T$, where $A^*$ is the optimal solution of (2.11). Replacing $\|BC\|_1$ by $f_\mu(B)$ in equation (2.9), we obtain

(2.12) \begin{equation*}\label{eq:proximal_fused_l1} \tilde{f}_{L1}(B) = \sum_k \left\{\frac{1}{n_k} \|y_k - X_k \beta_k\|^2_2\right\} + f_\mu(B)\end{equation*}

which is now continuously differentiable with gradient

(2.13) \begin{equation*}\label{eq:proximal_fused_l1_grad} \Delta \tilde{f}_{L1}(B) = \sum_k \left\{\frac{1}{n_k} X_k^T(X_K\beta_k-y_k)\right\} + f_\mu(B).\end{equation*}

Chen *and others* (2010) further show that $A^*=S(BC/\mu)$ where function S truncates each entry of $A^*$ to the range [-1,1] to ensure that $A^* \in \mathcal{Q}$. An upper bound $L_U$ of the Lipschitz constant $L$ can be derived as:

(2.14) \begin{equation*}\label{eq:lipschitz_bound} L_U = \max_k (\lambda_{max}(X_k^TX_k)) + \frac{\lambda^2+2*\gamma^2\max_{k\in V}d_k}{\mu}\end{equation*}

where $\lambda_{max}(M)$ is the largest eigenvalue of $M$ and $d_k = \sum_{k'}^K \tau_{k,k'}$.

With the derivation of the gradient in (2.13) and the Lipschitz bound in (2.14), we can now apply Nesterov’s method (Nesterov, 2005) for optimizing (2.12). The procedure is summarized in Algorithm 2. For more details on the proximal approach see Chen *and others* (2010).

**Algorithm 2:**
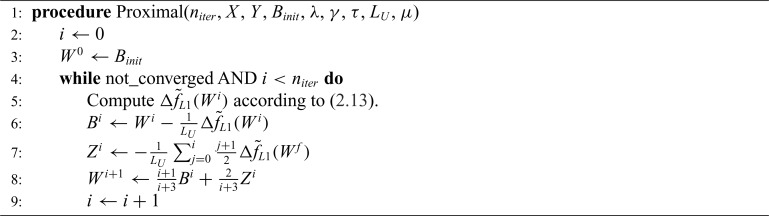
Proximal gradient optimization for the joint lasso

## 3. Simulation study

To test the performance of the proposed approach, we simulated data from a model based on characteristics of a recent drug response study, the Cancer Cell Line Encyclopedia (CCLE; Barretina *and others*, 2012). We base our simulation on real data in order to approximately mimic the correlation structure typical of human gene expression data; otherwise the set-up is generic and essentially the same issues would apply in many disease settings. To simulate data, we first estimated means and covariance matrices $\mu_k, \Sigma_k$ for each of $K=9$ subgroups (the eight cancer types with the latest sample sizes in CCLE plus a ninth for all other cancer types; covariances were estimated using the graphical lasso). For each group $k$, we then sampled features from the multivariate normal $\mathcal{N}(\mu_k, \Sigma_k)$. For a given total sample size $n$, subgroup sizes were consistent with those in the original data. We used a random subset of 200 gene expression levels (i.e. the dimensionality was fixed at $p=200$). This parametric approach allowed us to vary sample sizes freely, including the case of total $n$ larger than in the original dataset.

We are interested in the situation in which it may be useful to share information between subgroups. But we are also interested in investigating performance in settings that do not agree with our model formulation (the extreme cases being where subgroups are either entirely dissimilar or identical). Let $V = \{1 \ldots K \}$ be the set of subgroup indices (here, $K=9$). We set regression coefficients to be identical in a subset $V_0 \subseteq V$ of the subgroups, such that the size $K_0 = |V_0|$ of the subset governs the extent to which information sharing via the joint lasso could be useful. Specifically, if $K_0=K$, all subgroups have the same regression coefficients (i.e. favoring a pooled analysis using a single regression model) and at the other extreme if $K_0=1$ all groups have differently drawn coefficients. Intermediate values of $K_0$ give differing levels of similarity.

For a given value $K_0$, we defined membership of $V_0$ by considering the differences between the subgroup-specific models for the features. Specifically, we choose the $K_0$ groups that minimized the sum of symmetrised KL divergences between subgroup-specific models. A coefficient vector was then drawn separately for each subgroup $k \notin V_0$ and one, shared coefficient vector drawn for all $k \in V_0$. Each draw was done as follows. We first sampled a binary vector $b$ of length $p$ from a Bernoulli, i.e. $b_i \sim \mbox{Bernoulli}(0.1)$. Then we drew $\beta_i \sim \mathcal{N}_{trunc}(0,1)$ if $b_i = 1$ and set $\beta_i=0$ otherwise, where $\mathcal{N}_{trunc}(0,1)$ denotes a standard Normal with the interval $(-0.1,0.1)$ excluded (this is to ensure non-zero coefficients are not very small in magnitude). Note that in the case of $K_0=1$, all groups have separately drawn coefficients and the between-subgroup KL divergence plays no role.

We compare the joint lasso with pooled and subgroup-wise analyses. These are performed using classical lasso (we use the glmnet implementation) on respectively the whole dataset or each subgroup separately.

**Fig. 1.**
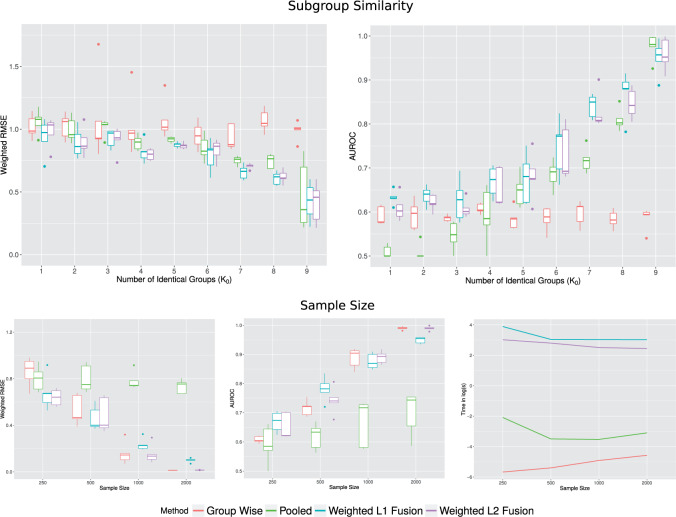
Simulated data performance. *Top row*: Varying $K_0$, where the number of subgroups is fixed at $K=9$ of which $K_0$ have shared coefficients in the underlying data-generating model (see text for details of simulation set-up). A smaller $K_0$ corresponds to less similarity between underlying subgroup-specific models, with $K_0=1$ representing the case where all subgroups have separately drawn coefficients, while $K_0=9$ represents an entirely homogenous model in which each subgroup has exactly the same regression coefficients. The total sample size is fixed at $n=250$. Left panel: Weighted root mean squared error (RMSE). RMSE is weighted by subgroup sizes. Right panel: Area under the ROC curve (AUROC) with respect to the true sets of active variables with non-zero coefficients). *Bottom Row*: Varying sample sizes, where the number of subgroups is fixed at $K=9$ of which $K_0=4$ have shared coefficients in the underlying data-generating model (see text for details of simulation set-up). Left panel: Root mean squared error (RMSE; weighted by subgroup sizes). Middle panel: Area under the ROC curve (AUROC); with respect to the true sets of active variables with non-zero coefficients. Right panel: Computational time taken in log seconds.

The top row of Figure 1 shows performance when varying the number $K_0$ of subgroups with shared coefficients, with the total number of samples fixed at $n=250$. Here, a smaller value of $K_0$ corresponds to less similarity between subgroup-specific coefficients in the underlying models. At intermediate values of $K_0$ the joint lasso offers gains over pooled and subgroup-wise analyses. This is because the pooled analyses are mis-specified due to the inhomogeneity of the data, while the subgroup-wise analyses, although correctly specified, must confront limited sample sizes since they analyze each subgroup entirely separately. In contrast, the joint lasso is able to pool information across subgroups, but also allows for subgroup-specific coefficients. Importantly, even at the extremes of $K_0=1$ (separately drawn coefficients for each subgroup) and $K_0=9$ (all subgroups have exactly the same coefficients), the joint lasso performs well. This demonstrates its flexibility in adapting the degree of fusion.

The bottom row of Figure 1 shows performance as a function of total sample size $n$. Here, the number of subgroups with identical coefficients is fixed at $K_0=4$. This gives a relatively weak opportunity for information sharing, since 5/9 groups have separately drawn coefficients. Since the true $\beta_k$’s are not identical, the pooled analysis is mis-specified and accordingly even at large sample sizes, it does not catch up with the other approaches. As expected, subgroup-wise analyses perform increasingly well at larger sample sizes. However, at smaller sample sizes the joint lasso shows some gains.

The $\ell_1$ and $\ell_2$ fusion approaches seem similar in performance. Note, however, that our $\ell_2$ implementation leverages the glmnet package and is more computationally efficient than the $\ell_1$ approach.

## 4. Alzheimer’s disease: prediction of cognitive scores

Here, we use data from the ADNI (Mueller *and others*, 2005) to explore the ability of the joint lasso to estimate regression models linking clinical and genetic features to disease progression, as captured by cognitive test scores. We emphasize that the purpose of this section is to illustrate the usefulness of the proposed method for data analysis and prediction. Prediction of AD and its progression remains an open topic and the wider applied themes are beyond the scope of this article.

In 2014, ADNI made a subset of its data available for a DREAM challenge (Allen *and others*, 2016) and we use these data here. The dataset consists of a total of $n=767$ individuals who were followed up over at least 24 months. Cognitive function was evaluated using the mini-mental state examination (MMSE). At baseline, individuals were classified as either cognitively normal (CN), early mild cognitive impairment (EMCI), late mild cognitive impairment (LMCI), or diagnosed with Alzheimer’s disease (AD). These form clinically-defined subgroups for our analysis. For the present analysis, we apply the method using the genetic data (single nucleotide polymorphisms or SNPs) and clinical profile only (we do not include neuroimaging data).

The goal is to predict the rate of decline in cognitive ability, as quantified by the slope of MMSE scores over a 24-month period. The total number of SNPs available is ${\sim}10^7$. Filtering by linkage disequilibrium reduces this to ${\sim}2 \times 10^6$. For computational and expository ease, we pre-selected 20 000 of this latter group that gave the smallest residuals when regressed with the clinical variables against responses in the training set. We note that the filtering step biases our analyses and estimates of out-of-sample error, but we emphasize that our goal here is not to propose a solution to the AD prediction problem but rather to compare the relative performance of various approaches (here all using the same fixed set of pre-selected features).

**Fig. 2.**
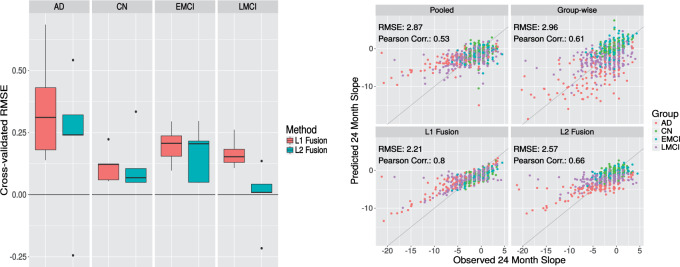
Alzheimers disease prediction results, ADNI data. Left panel: Box plots showing difference in RMSE of joint lasso with different fusion penalties compared with the pooled linear regression model (higher values indicate better performance by the joint lasso). [Subgroup-wise analysis performed less well than pooled and is not shown; boxplots are over 10-fold cross-validation.] Right panel: Scatter plots show predicted and observed 24-month slopes for each of the standard and joint lasso regression models. All predictions were obtained via 10-fold cross-validation.

Figure 2 (left) shows root mean squared error (RMSE) separately for each of the four subgroups. The joint lasso offers substantial gains compared with pooled and subgroup-wise analyses (the latter performed very badly and are not shown in the figure). The biggest gain with both fusion approaches is for the AD subgroup. A notable difference between $\ell_1$ and $\ell_2$ fusion is in the LMCI subgroup, where the $\ell_1$ fusion performs significantly better than pooled, while $\ell_2$ fusion only provides a marginal improvement. We also performed weighted fusion analysis (not shown), where the tuning parameters $\tau_{k,k'}$ were set using the distance between the means of each subgroup (in the space of genetic and clinical variables). Weighting did not appear to improve performance.

Figure 2 (right) shows scatter plots of predicted MMSE slopes versus the true slopes. The predictions were obtained in a held-out fashion via 10-fold cross-validation (CV), as were the RMSE and Pearson correlations shown. We see that predicted slopes from the $\ell_1$ approach better match the observed slopes, with the large improvement in Pearson correlation mostly driven by a few outliers in AD and LMCI. Overall the joint lasso improves on the pooled and group-wise approaches.

We further used the estimates of the effect sizes for the SNP data to perform a pathway enrichment analysis using the KEGG database (Kanehisa and Goto, 2000). The results are presented in Figure 1 of the supplementary material available at *Biostatistics* online. We show that increased fusion allows for the identification of common enriched pathways among the subgroups that would not be identified in a group-wise approach.

**Fig. 3.**
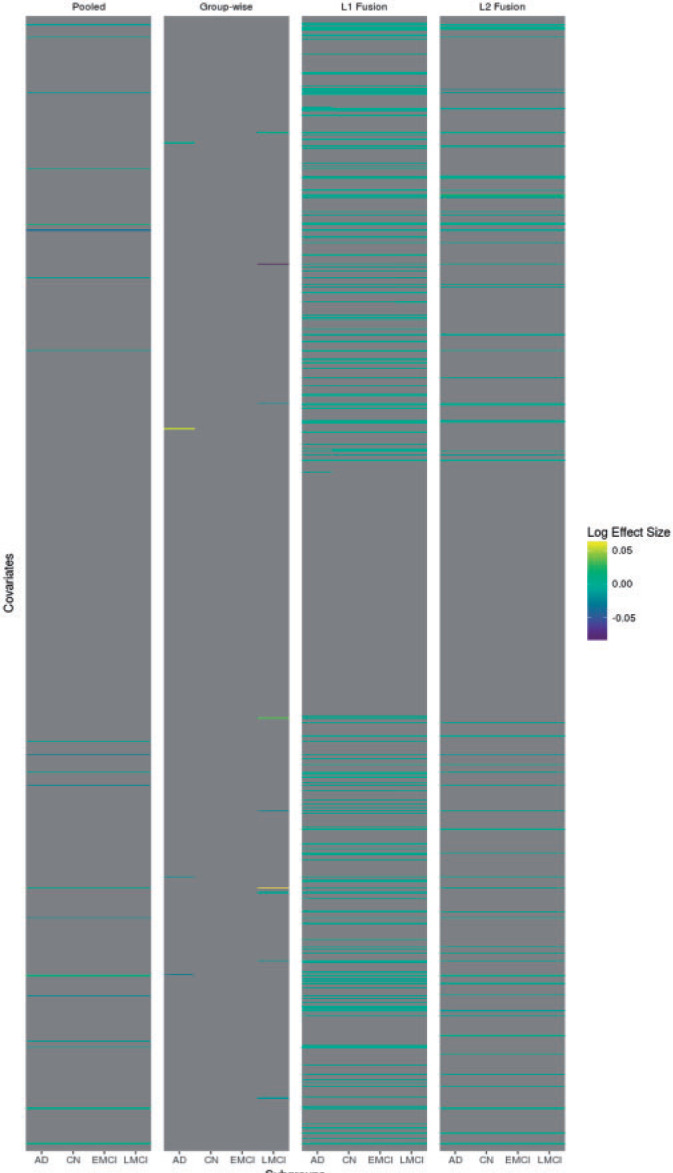
Alzheimer’s disease data, estimated regression coefficients. Heatmap showing estimated regression coefficients for a representative subsample of the SNPs. Absolute coefficients are thresholded at $e^{-2}$ to improve readability.

Figure 3 shows a comparison of the estimated regression coefficients themselves. The subgroup-wise approach is much sparser than the other methods, likely due to the fact that it must operate entirely separately on each (relatively small-sample) subgroup. In addition to loss of prediction power given finite training samples, this is another drawback of the group-wise approach, which is otherwise likely better specified than simple pooling. The pooled approach finds more influential variables but obviously there can be no subgroup-specificity. The joint lasso selects more variables than the subgroup-wise analysis, but there are many instances of subgroup-specificity in the estimates. The $\ell_1$ fusion penalty seems to have allowed for more differences between the subgroups than the $\ell_2$ penalty, with several instances where only one subgroup contains a non-zero coefficient. This likely explains the better performance on some of the outliers in AD and LMCI.

## 5. ALS: prediction of disease progression

ALS is an incurable neurodegenerative disease that can lead to death within three to four years of onset. However, about ten percent of patients survive more than 10 years. Prediction of disease progression remains an open question. We use data from the PROACT database, specifically data that were used in the 2015 DREAM ALS Stratification Prize4Life Challenge (data were retrieved from the PROACT database on June 22, 2015). As above, our aim is not to propose a solution to the prediction problem *per se* but rather to provide a case study exploring the use of the joint lasso in a moderate-dimensional, clinical data setting. In contrast to the Alzheimer’s example above, here the data are less high-dimensional and the subgrouping less clear cut (see below).

The data consist of observations from $n=2393$ patients. Each patient was enrolled in a clinical trial and followed up for a minimum of 12 months after the start of the trial. Disease progression is captured by a clinical scale called the ALS Functional Rating Scale (ALSFRS). The task is to predict the slope of the ALSFRS score from 3 to 12 months (after the start of the trial). For each patient, available information includes ALSFRS scores for the 0–3 month period, demographic information, and longitudinal measurements of clinical variables. We follow the featurization and imputation procedures devised by Mackay and Fang (see Küffner *and others*, 2015) and obtain a total of $p=615$ features.

Subgroups were defined as follows. The first subgroup consists of patients with disease onset before the start of the trial. The second subgroup consists of patients for whom onset was after the start of the trial and who have negative ALSFRS slope. The third subgroup of patients also had onset after the start of the trial but positive ALSFRS slope. Thus, the subgroups reflect severity of onset.

**Fig. 4.**
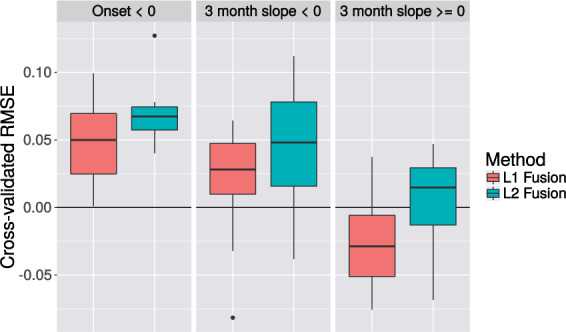
ALS prediction results. Box plots showing difference in RMSE of the joint lasso compared with the pooled linear regression model (higher values indicate better performance by the joint lasso). [Subgroup-wise analysis performed less well than pooled and is not shown; boxplots are over 10-fold cross-validation.]

Figure 4 shows (held-out) RMSEs by subgroup; we see that the largest improvement in prediction performance is in subgroup 1. The joint lasso approach leads to a modest improvement. Overall, the $\ell_2$ approach seems to perform slightly better than the $\ell_1$ approach. In particular, there is a slight decrease in performance compared to the pooled method for $\ell_1$ fusion in subgroup 3. The difference between weighted and unweighted fusion is negligible and was not included in the figure.^2^

## 6. Prediction of therapeutic response in cancer cell lines

The Cancer Cell Line Encyclopedia (CCLE; Barretina *and others*, 2012) is a panel of 947 cancer cell lines with associated molecular measurements and responses to 24 anti-cancer agents. Here, we use these data to explore group-structured regression. We treat the area above the dose-response curve as the response and use expression levels of ${\sim}$20 000 human genes as features. We treat the cancer types as subgroups $k$. After discarding cell lines with missing values, we arrive at $n\sim 500$ samples.

**Fig. 5.**
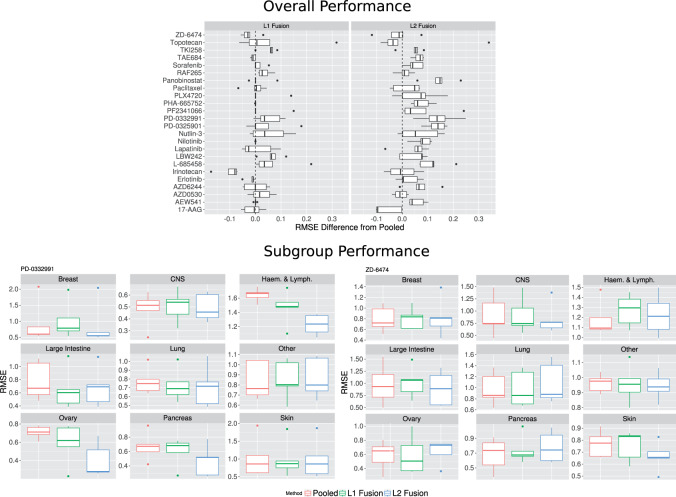
Cancer cell line therapeutic response prediction. *Top panel*: Difference in weighted RMSE between the joint lasso with L1 and L2 fusion penalty and a pooled analysis. Results shown over 24 responses (anti-cancer agents) using data from the Cancer Cell Line Encyclopedia (CCLE); the dashed vertical lines at zero indicate no difference, boxplots to the right indicate improvement (lower RMSE) over pooled. *Bottom panels*: Cancer cell line therapeutic response prediction, broken down by subgroup (cancer type) for agents PD-0332991 and ZD-6474.

Figure 5 (top) shows results over all 24 responses (anti-cancer agents). We observe that for most responses the joint lasso with $\ell_2$ fusion approach shows either improved or similar prediction performance to pooled in terms of RMSE (weighted by subgroup size). In contrast, the $\ell_1$ fusion approach only shows improved performance in a small number of drugs; for most drugs, the performance is indistinguishable from the pooled approach. This indicates that $\ell_1$ fusion over-regularizes in this example, forcing all coefficients to be the same, and reverting to the pooled model. Weighted fusion shows a similar performance to unweighted fusion (not shown).

Figure 5 (bottom) shows results broken down by subgroup for two examples (responses PD-0332991 and ZD-6474). In the former case, the joint lasso largely outperforms pooled and subgroup-wise analyses. In the second, pooled is the best performer, although the joint lasso performance is similar in most subgroups.

## 7. Conclusions

Advances in data acquisition and storage are changing the nature of biomedical studies. Datasets are often heterogenous, with samples spanning multiple disease types (or other biological or medical contexts) that may be related but also expected to have differences with respect to underlying biology. Large, heterogeneous data give opportunities to study similarities and differences between related pathologies and to gain power in high-dimensional estimation by pooling information across larger sets of samples. Indeed, many large datasets should arguably be thought of as comprising several smaller datasets, that have similarities but that cannot be assumed to be identically distributed. Statistically efficient regression in such settings will require ways to pool information where useful to do so, whilst retaining the possibility of subgroup-specific parameters and structure (such as sparsity patterns). We proposed a penalized likelihood approach called the joint lasso for high-dimensional regression in the group-structured setting that provides group-specific estimates with global sparsity and that allows for information sharing between groups.

We proposed two variants of the joint lasso, with an $\ell_1$ and $\ell_2$ fusion penalty respectively. The main theoretical difference between the two approaches is that the $\ell_1$ fusion approach has a discontinuity at $\beta_k=\beta_k'$, which encourages “sparsity of the differences” (Tibshirani *and others*, 2005), or in other words, encourages the coefficients in different subgroups to be exactly the same. In practice, we have found that in our simulation studies there was little difference in predictive performance between the two types of penalty, while the real data applications varied as to which variant performed better. In the ALS and CCLE examples, the $\ell_2$ approach performed better in terms of improvement over the pooled approach, while in the Alzheimer’s dataset, the $\ell_1$ approach lead to a greater improvement. We conclude that the choice of penalty will be data-dependent, and will be influenced by whether the $\ell_1$ prior assumption of equality of coefficients between pairs of subgroups makes sense for the problem setting. In cases where this is not known, we would recommend starting by applying the $\ell_2$ approach, which is less computationally expensive due to the absence of discontinuities in the fusion penalty.

In any given application, even when there are good scientific reasons to suspect differences in regression models between subgroups, it is hard to know in advance whether the nature of any differences is such that a specific kind of joint estimation would be beneficial. For example, if sample sizes are small and groups only slightly different, pooling may be more effective, or if the groups are entirely different, fusion of the kind we consider may not be useful. This means that in practice, either simple pooling or subgroup-wise analysis may be more effective than the joint lasso. In our approach, the tuning parameter $\gamma$ (set by cross-validation) determines the extent of fusion in a data-adaptive manner, and we saw in several examples that this appears successful in giving results that are at worst close to the best of pooling and subgroup-wise analyses. For settings with widely divergent subgroup-specific sample sizes $n_k$, it may be important to allow tuning parameters to depend on $n_k$ (we did not do so) and to consider alternative formulations that allow for asymmetric fusion.

An appealing feature of the joint lasso is that it allows for subgroup-specific sparsity patterns and parameter estimates that may themselves be of scientific interest. We discussed point estimation, but did not discuss uncertainty quantification for subgroup-specific estimates. A number of recent papers have discussed significance testing for lasso-type models (see e.g. Wasserman and Roeder, 2009; Lockhart *and others*, 2014; Städler and Mukherjee, 2016) and we think some of these ideas could be used with the models proposed here.

## 8. Software availability

The R code used for the experiments in this paper has been made available as R package fuser on CRAN: https://cran.r-project.org/web/packages/fuser. Scripts for reproducing the results in this paper can be obtained at: http://fhm-chicas-code.lancs.ac.uk/dondelin/SubgroupFusionPrediction.

## Supplementary Material

kxy035_Supplementary_Information

## Footnotes

^1^It would be possible to reformulate the first part of the objective in matrix form $Y_{diag} - X_{diag}B_{diag}$ by defining $X_{diag}$ as a block-diagonal matrix as in Section 2.5.1, defining $B_{diag}$ as a $pK \times K$ matrix with $\beta_k$ along the diagonals and similarly $y_{diag}$ as an $n \times K$ matrix with $y_k$ along the diagonals; however, this formulation is neither practical nor intuitive, and the gain in notational simplicity is negligible.

^2^This dataset is different from the one reported in (Küffner *and others*, 2015), with larger variance in the slopes, and so RMSE values are not directly comparable; however, we note that performance for our methods compares favorably with that reported in the reference.
