# Supplementary material for: The joint lasso: high-dimensional regression for group structured data
Source: Biostatistics. 2018 Sep 5;21(2):219–35. doi: 10.1093/biostatistics/kxy035 (PMC7868060; doi:10.1093/biostatistics/kxy035)
Supplement: kxy035_Supplementary_Information [file kxy035_supplementary_information.pdf]

Supplementary Information for "The joint lasso: High-dimensional regression for group structured data"

Frank Dondelinger and Sach Mukherjee

1 Pathway Analysis of ADNI Results

1.1 Methodology

In order to demonstrate further characteristics of our method for information sharing across subgroups, we perform a pathway enrichment analysis. The aim is to determine the number of significantly enriched pathways in the ADNI dataset based on the SNP effect sizes in the multiple regression on the outcome (cognitive decline). We will not recap the penalized regression framework, which is described in the main text, but we will describe how p-values for pathway enrichment are derived based on the ranking of effect sizes.

First, a list of pathways in Homo sapiens, the proteins active in them, and the genes coding for these proteins is retrieved from the Kegg database [1]. In order to calculate enrichment, we require a ranking of the genes in the pathway, which means that we need to associate SNPs with genes, which is done using the `bitr` function of the `clusterProfiler` package [4]. For simplicity, if there is more than one SNP associated with a given gene, we retain only the SNP with the largest effect size. The genes are then ranked according to absolute effect size of the associated SNPs; genes with no associated SNPs are assigned the lowest rank.

Finally, we use the `wilcoxGST` function in `limma` [3] to run an enrichment test based on the Wilcoxon rank-sum [2]. Only pathways with p-value ≤ 0.05 after Benjamini-Hochberg correction for multiple testing are considered significant.

1.2 Results

Although it would be possible to visualize the significantly enriched pathways, we choose not to do so for two reasons: 1) the sample size of the ADNI dataset, while large enough for the purposes of demonstrating improvements in prediction, is not large enough to allow for any reliable validation of our results on a held-out dataset, creating the risk of potentially spurious results for any given patway; 2) the procedure for choosing a subset of SNPs for our analysis creates the risk of biased results. While this does not affect the comparison of prediction methods, it does create a danger of over-interpreting results that may not generalize to other datasets.

Instead, we use the pathway analysis to investigate the characteristics of our method under increasing fusion of subgroup parameters, as regularized by the γ hyperparameter. Figure 1 shows the evaluation of number of significantly enriched pathways as γ increases. The λ parameter is determined by cross-validation as before. We note that for small γ (minimal information sharing), few pathways are identified and they differ per group. As γ increases, additional common pathways are identified. For very large γ, the number of significant pathways decreases again; this is a consequence of the tradeoff between γ and λ; overly large γ values will increase the sparsity of β_k vectors of linear parameters for each group k. This is an interesting property of our method, but does not adversely impact estimation as long as γ is chosen by cross-validation.

2 Effect of Subgroup Weighting

The model proposed in the paper has the following criterion:

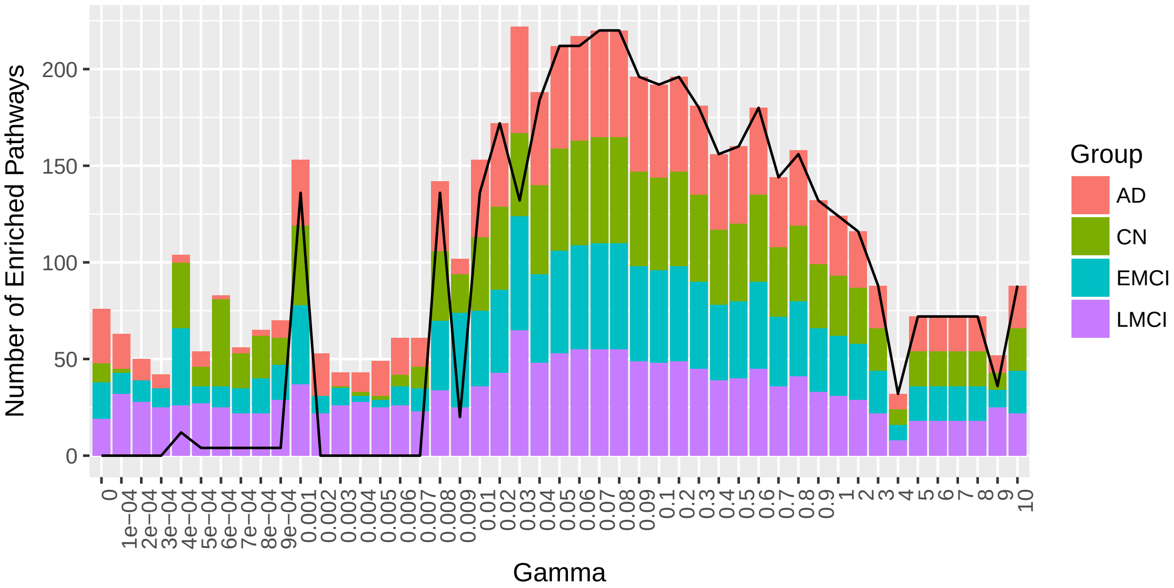

Figure 1: Number of significant patways in each of the four subgroups of the ADNI dataset (AD - Alzheimer's disease, EMCI - early mild cognitive impairment, LMCI - late mild cognitive impairment, CN - cognitive normal). Bar plots show number of significant pathways for a given value of γ, while the black line shows the number of pathways that are deemed significant in all subgroups (scaled by the number of subgroups).

$$\hat{B} = \underset{B=[\beta_1 \ldots \beta_k]}{\arg\min} \sum_{k=1}^{K} \left\{ \frac{1}{n_k} \|y_k - X_k\beta_k\|_2^2 + \lambda\|\beta_k\|_1 + \gamma \sum_{k'>k} \tau_{k,k'} \|\beta_k - \beta_{k'}\|_2^2 \right\}$$

It would be possible to define a very similar model which excludes the $\frac{1}{n_k}$ terms, i.e.:

$$\hat{B} = \underset{B=[\beta_1 \ldots \beta_k]}{\arg\min} \sum_{k=1}^{K} \left\{ \|y_k - X_k\beta_k\|_2^2 + \lambda\|\beta_k\|_1 + \gamma \sum_{k'>k} \tau_{k,k'} \|\beta_k - \beta_{k'}\|_2^2 \right\}$$

The difference is that the first model weights the sum of squared errors by the number of samples in each subgroup (in effect using the mean sum of squared errors), while the second model does not. The unweighted approach can lead to sub-optimal performance in pathological cases where the group sizes differ drastically. To demonstrate this, we performed a small simulation study (Figure 2) with three subgroups, where two share the same coefficient vector β_1 and a third divergent subgroup has coefficient vector β_2. We set $\lambda = 0$ and $\gamma = 1$ for the unweighted model in a deliberate misspecification to force fusion of the coefficients. In the subgroup weighting model, we set $\gamma = k/n$, which ensures comparable fusion penalization between the two models.

The simulation study shows that estimation of the coefficients is unaffected by subgroup weighting as long as the sample size of the divergent subgroup is similar to the size of the two other subgroups. However, when the divergent subgroup is much larger than the other two subgroups, subgroup weighting leads to an improvement in the estimation error.

References

[1] Minoru Kanehisa and Susumu Goto. KEGG: Kyoto encyclopedia of genes and genomes. *Nucleic acids research*, 28(1):27–30, 2000.

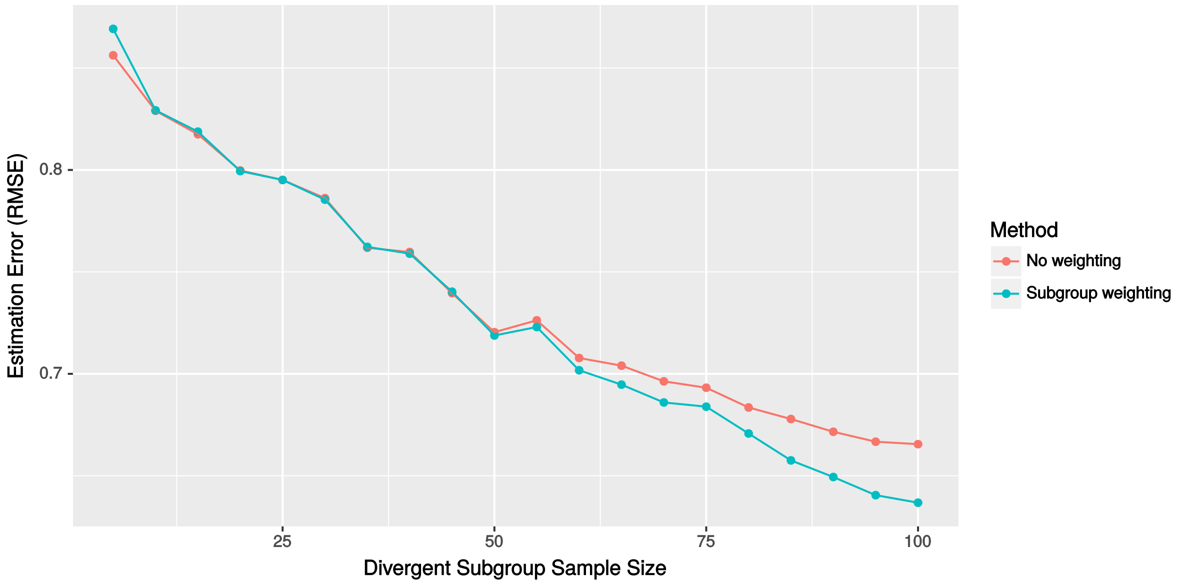

Figure 2: Simulation of the effect of subgroup weighting in a pathological dataset consisting of three subgroups; groups 1 and 2 of sample size $n = 25$ and divergent group 3 of varying sample size. Data for group 1 and 2 are generated from a linear regression model with coefficient vector β_1 of size $p = 25$, while data for the divergent group is generated from a different linear regression model with coefficient vector β_2. In a deliberate misspecification, we set λ and γ such that fusion of the coefficients is enforced. As the sample size of the divergent group increases, the RMSE of the fused lasso estimate of the coefficients should decrease. We observe that under the model weighted by the subgroup size, this happens more quickly than under the unweighted model once the imbalance among the subgroup sizes becomes sufficiently large.

[2] Joëlle Michaud, Ken M Simpson, Robert Escher, Karine Buchet-Poyau, Tim Beissbarth, Catherine Carmichael, Matthew E Ritchie, Frédéric Schütz, Ping Cannon, Marjorie Liu, et al. Integrative analysis of RUNX1 downstream pathways and target genes. *BMC genomics*, 9(1):363, 2008.

[3] Gordon Smyth. Limma: linear models for microarray data. *Bioinformatics and computational biology solutions using R and Bioconductor*, pages 397–420, 2005.

[4] Guangchuang Yu, Li-Gen Wang, Yanyan Han, and Qing-Yu He. clusterProfiler: an R package for comparing biological themes among gene clusters. *Omics: a journal of integrative biology*, 16(5):284–287, 2012.
